# The BioSAXS laboratory of the ITACA.SB Research Infrastructure

**DOI:** 10.1107/S1600576726007132

**Published:** 2026-08-24

**Authors:** Francesco Scattarella, Davide Altamura, Vincenzo Mangini, Teresa Sibillano, Erika Manicone, Rocco Lassandro, Liberato De Caro, Gemma Newby, Cinzia Giannini

**Affiliations:** a Istituto di Cristallografia Consiglio Nazionale delle Ricerche via Amendola 122/O Bari 70126 Italy; b Dipartimento di Chimica, Università degli Studi di Bari, Via Edoardo Orabona 4, Bari, 70126, Italy; c Xenocs SAS, Allée du Nanomètre, Grenoble, 38000, France; Universität Duisburg-Essen, Germany

**Keywords:** small-angle X-ray scattering, SAXS, wide-angle X-ray scattering, WAXS, scanning microscopy, size-exclusion chromatography SAXS, SEC-SAXS, ultra-small-angle X-ray scattering, USAXS

## Abstract

We describe a state-of-the-art X-ray scattering laboratory, within the Research Infrastructure ITACA.SB (Potentiating the Italian Capacity for Structural Biology Services in Instruct-ERIC), designed for multi-scale structural characterization of biological specimens and advanced materials with small- and wide-angle X-ray scattering (SAXS/WAXS) techniques: WAXS to probe atomic and molecular organization, and SAXS to characterize morphological and structural features at the nanoscale. The system enables (i) the concurrent acquisition of SAXS/WAXS data, (ii) the execution of scanning SAXS/WAXS microscopy, and (iii) the collection of ultra-small-angle X-ray scattering, batch BioSAXS and size-exclusion chromatography SAXS data. To demonstrate the system’s versatility, we investigated the hierarchical complexity of both biological specimens and synthetic/natural fibers. We also report on analysis of well known proteins and micelles.

## Abstract 2

We present a versatile state-of-the-art X-ray scattering laboratory within the ITACA.SB Research Infrastructure, enabling multi-scale structural characterization of complex biological specimens and advanced materials through integrated small- and wide-angle X-ray scattering (SAXS/WAXS) techniques. The platform supports simultaneous SAXS/WAXS acquisition, scanning microscopy, ultra-SAXS and size-exclusion chromatography SAXS measurements.

## 1. Introduction

For decades, high-flux X-ray microfocus sources were exclusively available at synchrotron radiation facilities, where the combination of exceptional brilliance and tunable beam properties enabled experiments far beyond the reach of conventional laboratory systems. Dedicated beamlines at world-class centers – such as the European Synchrotron Radiation Facility (ESRF-EBS) (Bruno *et al.*, 2024 ▸), Diamond Light Source, PETRA IV (Schroer *et al.*, 2018 ▸), and fourth-generation diffraction-limited storage rings (Eriksson *et al.*, 2014 ▸) including MAX IV (Robert *et al.*, 2023 ▸) and Sirius (Liu *et al.*, 2019 ▸) – routinely deliver micro- and nanofocused beams with dimensions ranging from a few micrometres down to tens of nanometres. These facilities have continuously pushed the technological frontier of X-ray science (Mino *et al.*, 2018 ▸).

Current developments at synchrotron facilities concentrate on three major directions. First, extreme focusing capabilities are achieved through advanced X-ray optics, including compound refractive lenses and Kirkpatrick–Baez mirror systems, enabling nanofocus beams with unprecedented spatial resolution and coherence (Salditt & Osterhoff, 2020 ▸). Second, hybrid and multimodal approaches are increasingly integrated within a single experimental platform. Scanning small- and wide-angle X-ray scattering (SAXS/WAXS) is now combined with complementary techniques such as X-ray fluorescence (Paris *et al.*, 2007 ▸) for elemental mapping, allowing simultaneous structural, chemical and morphological characterization. Third, high-speed detection technologies have revolutionized data acquisition. State-of-the-art pixel-array detectors, such as EIGER (Radicci *et al.*, 2012 ▸) and JUNGFRAU (Redford *et al.*, 2018 ▸) systems, operate at kilohertz frame rates with single-photon sensitivity, enabling time-resolved studies of dynamic processes, *in situ* reactions and mechanical deformation with sub-millisecond temporal resolution.

Despite these remarkable capabilities, access to synchrotron facilities remains highly competitive, governed by peer-reviewed proposal cycles and limited beamtime allocations. Consequently, the development of advanced laboratory-based X-ray instrumentation has been a major focus over the past decade. The performance gap between synchrotron and laboratory systems has significantly narrowed, largely due to breakthroughs in source brilliance, optical design and single-photon-counting detectors operating in vacuum. Modern laboratory platforms, such as the Xeuss 3.0 HR (Xenocs SAS, Grenoble, France, https://www.xenocs.com) equipped with liquid metal-jet sources (*e.g.* Excillum technology, Stockholm, Sweden, https://www.excillum.com/) or next-generation high-brilliance rotating anodes, now approach what can reasonably be termed ‘synchrotron-class’ performance for many structural investigations. Examples are the investigation of green crop biomass proteins for their potential in stabilizing emulsions (Müller *et al.*, 2025 ▸) conducted at Heinz Maier-Leibnitz Zentrum, and studies on the crosslinking between levodopa and lysine carried out at Lund University (Cole *et al.*, 2025 ▸).

Although laboratory beam sizes typically are ∼100 µm – larger than synchrotron nanobeams – the dramatic increase in photon flux and stability enables high-quality structural and morphological characterization in a home-laboratory environment, with greater flexibility and continuous access. Within this context, SAXS and WAXS, with their counterparts in reflection mode grazing-incidence SAXS/WAXS (GISAXS/GIWAXS), continue to represent the gold standard for non-invasive, multi-scale structural analysis. SAXS probes electron-density fluctuations in the 1–100 nm range, providing quantitative information on nanoscale morphology, hierarchical organization and supramolecular periodicities. WAXS complements this by accessing atomic- and molecular-scale order, revealing crystallinity, phase composition, intermol­ecular packing and unit-cell parameters. The combination of SAXS and WAXS thus bridges length scales from Å-level molecular structure to mesoscale architecture within a single experimental framework.

The current frontier of X-ray structural science lies also in scanning SAXS/WAXS microscopy (Gourrier *et al.*, 2007 ▸; Liu & Makowski, 2022 ▸). By raster-scanning a specimen through a microfocused beam and collecting a diffraction pattern at each pixel, it becomes possible to generate spatially resolved maps of structural parameters across millimetre/centimetre-scale areas with micrometre resolution. This approach transforms scattering from a volume-averaging technique into a quantitative imaging modality. Structural metrics, such as intermolecular spacing, degree of orientation, crystallinity or periodicity, can be statistically evaluated across heterogeneous materials, revealing spatial gradients, defects and hierarchical transitions (Giannini *et al.*, 2020 ▸).

Scanning SAXS/WAXS is particularly central to the emerging field of materiomics (Cranford *et al.*, 2013 ▸), which seeks to correlate hierarchical structure with macroscopic function in both biological and engineered systems. Natural tissues, such as collagen-rich connective matrices and cellulose-based plant structures, exhibit complex multi-scale organization that directly governs their mechanical, optical and transport properties. Similarly, advanced synthetic materials and architected metamaterials rely on precisely engineered hierarchical architectures to achieve tailored performance. The ability to quantitatively map structure–function relationships across multiple length scales is therefore transformative.

Importantly, what was once an exclusive capability of synchrotron beamlines is increasingly feasible in advanced laboratory settings. The combination of high-brilliance sources, optimized focusing optics and fast photon-counting detectors now allows laboratory-based scanning SAXS/WAXS (SWAXS) experiments with sufficient signal-to-noise ratio and throughput for systematic studies (Altamura *et al.*, 2012 ▸).

Beyond materials science and hierarchical tissue analysis, SAXS has become a cornerstone technique in structural biology under the framework of BioSAXS. Unlike crystallography, which requires ordered crystals, BioSAXS enables the investigation of macromolecules directly in solution under near-physiological conditions (Mertens & Svergun, 2010 ▸). This capability is particularly valuable for proteins, nucleic acids and macromolecular complexes that are flexible, partially disordered or difficult to crystallize, as it enables structural characterization under more physiologically relevant conditions.

While scanning SWAXS applications prioritize extreme beam focusing to achieve high spatial resolution, BioSAXS has distinct requirements including a high photon flux to characterize dilute macromolecular solutions and exceptional beam stability for precise background subtraction. In this regard, synchrotron facilities maintain a clear advantage for time-resolved studies, whereas laboratory-based instruments provide essential accessibility for preliminary sample screening with much lower radiation damage probability.

BioSAXS provides low-resolution structural information in the 1–100 nm range, yielding parameters such as the radius of gyration (Rg) and maximum particle dimension (Dmax) as well as allowing molecular weight estimation and overall shape reconstruction through *ab initio* modeling. Importantly, the technique captures ensemble-averaged conformations in solution, making it uniquely suited for studying conformational changes, ligand binding, oligomerization states and dynamic equilibrium between structural states. For intrinsically disordered proteins or multi-domain systems connected by flexible linkers, BioSAXS often represents the only experimental method capable of probing global architecture without imposing artificial constraints.

A major evolution in this field is the integration of size-exclusion chromatography with SAXS, commonly referred to as SEC-SAXS (David & Pérez, 2009 ▸). In conventional BioSAXS experiments, sample heterogeneity, such as aggregation, oligomeric mixtures or degradation products, can compromise data quality and interpretation. SEC-SAXS addresses this limitation by coupling an in-line chromatographic separation step directly to the SAXS flow cell. As the sample elutes from the chromatography column, scattering data are continuously collected, allowing isolation of monodisperse fractions in real time. This approach ensures that structural analysis is performed on homogeneous species, significantly improving reliability, reproducibility and interpretability.

SEC-SAXS has become particularly important for studying weakly interacting complexes, transient assemblies and systems prone to aggregation. It enables accurate molecular weight determination, discrimination between oligomeric states and detection of subtle conformational transitions. When combined with complementary techniques such as multi-angle light scattering, dynamic light scattering or UV absorbance, SEC-SAXS provides a comprehensive biophysical characterization pipeline.

Recent advances in detector sensitivity, automation and data-processing pipelines have further streamlined BioSAXS and SEC-SAXS workflows. High-throughput robotic sample changers, standardized quality-control metrics and automated modeling software now allow routine structural screening of biomolecules (Tuukkanen *et al.*, 2017 ▸).

In the broader context of multi-scale structural science, BioSAXS and SEC-SAXS extend the reach of scattering techniques from hierarchical materials and tissues down to isolated biomolecular systems. Together with WAXS and scanning SWAXS microscopy, they form a comprehensive toolkit spanning atomic structure, macromolecular organization in solution, supramolecular assembly and spatially resolved tissue architecture. This continuum of length scales underscores the versatility of X-ray scattering as a unifying methodology for investigating structure–function relationships across biology and materials science.

In this work, we describe in Section 2 a facility installed at the Institute of Crystallography, National Research Council in Bari, within the ITACA.SB Research Infrastructure (Potentiating the Italian Capacity for Structural Biology Services in Instruct-ERIC, https://www.itaca-sb.it/), designed for multi-scale structural characterization of biological specimens and advanced materials with SAXS/WAXS techniques. Its performance is presented in Section 3 through a series of representative case studies, including measurements on well characterized proteins (lysozyme, carbonic anhydrase II, human insulin, bovine serum albumin and apoferritin), micellar systems (PS20, VitE-TPGS) and, finally, hierarchically complex materials, primarily fiber-based systems. In Section 4, we discuss future perspectives and potential developments, with particular emphasis on upcoming applications and opportunities enabled by the platform.

## 2. Instrument description

**Figure 1:**
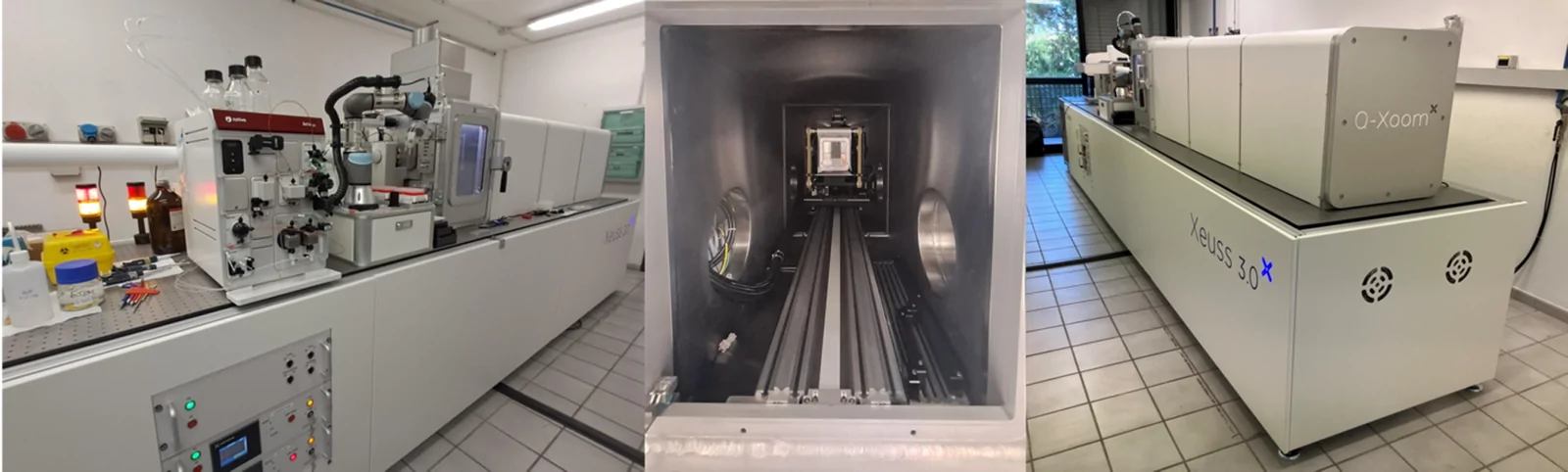
Views of the Xeuss 3.0 HR system, installed at the Institute of Crystallography, National Research Council in Bari, with a close-up of the motorized vacuum flight tube, showing the detector’s travel path (center).

The Xeuss 3.0 HR (high resolution) system by Xenocs is a state-of-the-art laboratory X-ray scattering platform designed for multi-scale structural characterization (Fig. 1 ▸).

**Figure 2:**
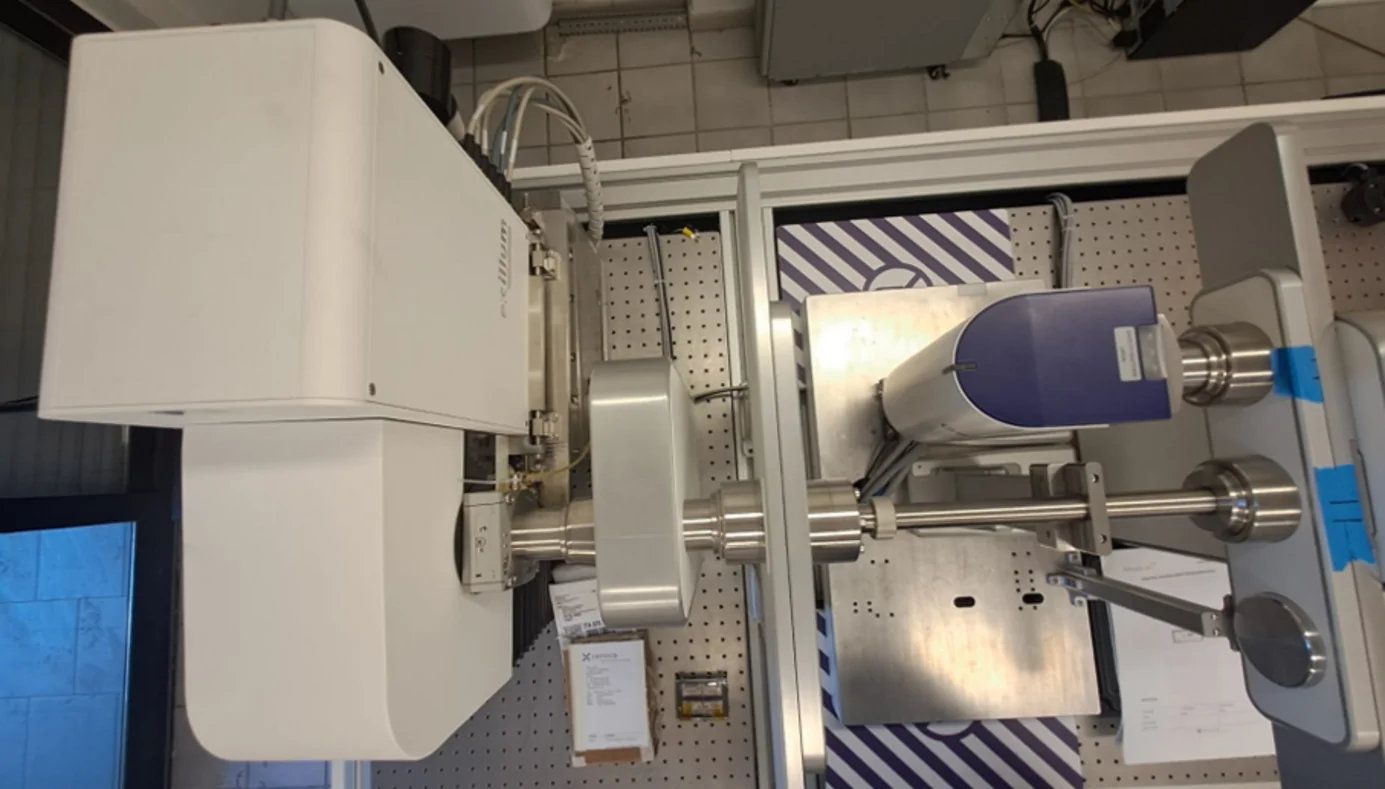
Top view of the motorized dual-source configuration with a Ga Excillum liquid metal-jet source (white) and a Cu GENIX^3D^ microfocus source (blue) integrated in the Xeuss 3.0 HR system.

The instrument features a motorized dual-source configuration (Fig. 2 ▸), integrating a high-brilliance Ga liquid metal-jet source (Excillum Metaljet D2+, λ = 1.34144 Å) and a Cu microfocus source (GENIX^3D^, λ = 1.54189 Å), allowing for optimized wavelength selection based on sample absorption and scattering requirements. Considering the proximity of the two emission energies, the primary advantage of the dual-source configuration in the Xeuss 3.0 is not to provide broad energy tunability but rather to offer a choice between different beam brilliances and flux profiles. The Ga liquid metal-jet source delivers significantly higher brilliance than the Cu microfocus source, with a photon flux more than four times higher than that of Cu. This is critical for BioSAXS measurements of dilute solutions, advisable for accelerating data acquisition in scanning SWAXS mapping and mandatory for ultra-small-angle X-ray scattering (USAXS) data. A fully automatic collimation system with adjustable slits enables selection of the beam size on the sample from predefined options, ranging from a minimum of 0.15 × 0.15 mm (ultra-high-resolution mode) to a maximum of 8 × 8 mm (full open mode).

The system is equipped with an EIGER2 R 1M detector (https://www.dectris.com/en/), which operates within a motorized vacuum flight tube. The sample-to-detector distance (SDD) can be continuously adjusted from 42.5 to 1800 mm, enabling a seamless transition between WAXS and SAXS regimes. For USAXS applications, the instrument utilizes a monochromatic beam from a multi-bounce Bartels Si(111) crystal monochromator (channel-cut) coupled with a four-bounce Si(111) crystal analyzer, pushing the detection limits to resolve hierarchical structures at the micrometre scale. A second four-axis motorized SWAXS module with an EIGER2 R 500K detector is available to collect WAXS data, concurrently to SAXS (with an EIGER2 R 1M detector at the 1800 mm position, or at a shorter distance but compatible with the simultaneous presence of EIGER2 R 500 K), covering 1/8th of the Ewald sphere in forward scattering and achieving a 2θ range for scattering from 7° to 90°. The rotation travel in the perpendicular plane is 90° for in-plane scattering or collection at high azimuth angles. It includes vertical translation to move the detector out of the chamber and control detector inclination.

The Xeuss 3.0 can operate in a variety of setups. To enhance usability, the system has been designed for rapid and straightforward switching between these operational configurations, common to all setups. Some operate fully automatically, while others allow for user intervention where required. Complete USAXS/SAXS/WAXS experiments can be conducted in a fully automated manner using the macro mode of the control software. Transitions between sample environments and experimental options have been simplified through ready-to-connect interfaces. Upon connection and start-up, the system automatically recognizes the selected sample environment. Typical changeover times between standard measurements using different static sample holders are less than 1 min while transitions involving *in situ* controlled devices are in the range of a few minutes, excluding venting and evacuation times. Exposure times depend on the nature of the sample being measured, ranging from a few seconds for strongly scattering systems to several hours for weakly scattering samples such as dilute protein solutions. For data reduction, radial averaging is performed automatically by the system using *XSACT 2.10* (Xenocs, 2023 ▸), Xenocs’ advanced SAXS/WAXS data analysis software, which among its various features offers automatic 2D-to-1D reduction (also in absolute intensity units) and reciprocal-space visualization.

Detector calibration is performed using standard reference materials. According to the instrument specifications, for the EIGER2 R 1M detector, lanthanum hexaboride (LaB_6_) is used to calibrate the SDD at around 60 mm. This calibration is then valid for the entire range of the detector translation since the motion along the axis is very reproducible and verified on instrument installation. Silver behenate can be used to check the longer distances between 60 and 1800 mm, if required. For the EIGER2 R 500 K detector, LaB_6_ is also employed for calibration over all accessible positions and detector inclinations. The calibration procedure is typically carried out once and applied to all scattering geometries and measurement configurations. Although the system is calibrated and automatically updated when the SDD is changed and adapted to different sample environments, it is always good practice to verify the calibration of the scattering geometry for each set of samples.

Advanced biological characterization is supported by the BioCube System and a fully integrated SEC-SAXS setup. The latter is coupled with an ÄKTA go (https://www.cytivalifesciences.com/en/gb) protein purification system, allowing for the real-time structural analysis of monodisperse macromolecular solutions.

**Figure 3:**
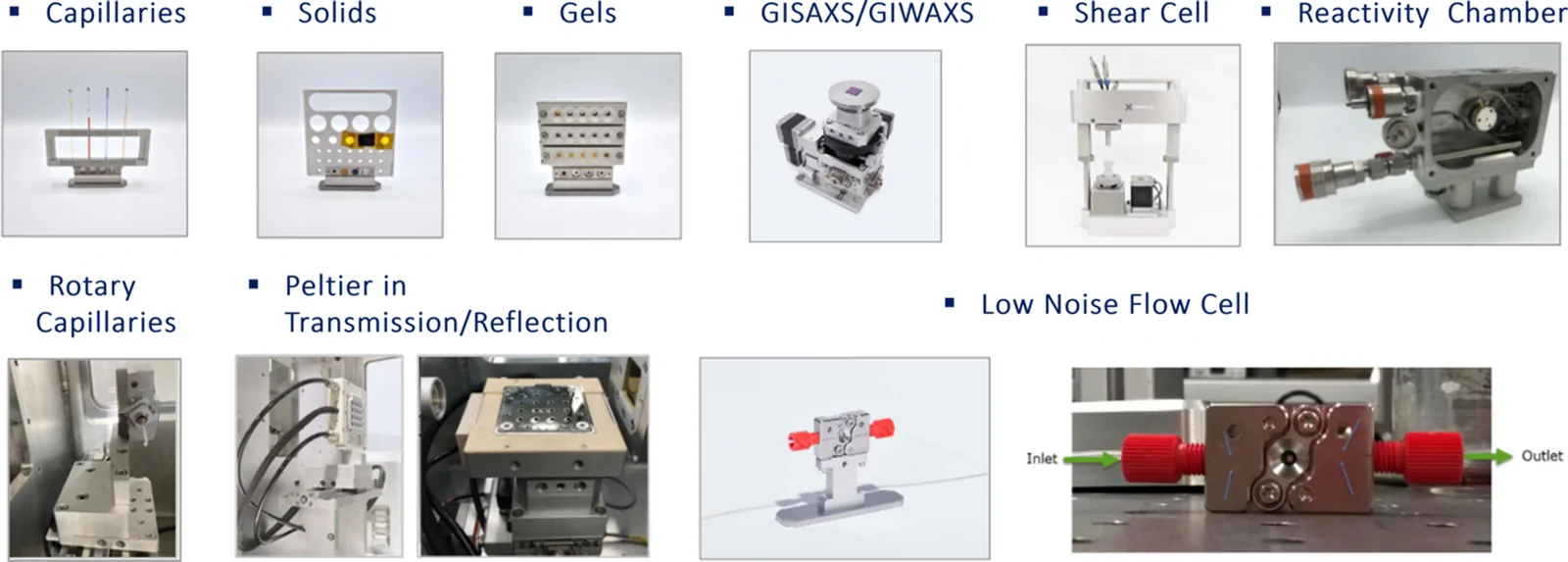
Sample environment/holders available at the BioSAXS facility.

Furthermore, the instrument is complemented by a versatile range of sample holders (Fig. 3 ▸) for diverse environmental conditions and a virtual detector capability, which facilitates extended *q*-range coverage in a single measurement sequence.

The system is also equipped with the following:

(i) Low-noise flow cell (LNFC): vacuum-compatible low scattering flow cell for measurements on liquids. Manual injection can be performed with large volumes as well as with volumes down to a few tens of µl with a no-dead-volume solution from syringe to cell. The theoretical volume of the cell is 7 µl, with a nominal cell thickness of 1.1 mm. However, to ensure complete and proper filling, a sample volume of approximately 15–20 µl is recommended. Minor variations in cell thickness may occur due to manufacturing tolerances of the sealing components. Therefore, the actual sample thickness is determined experimentally using the X-ray absorption of water, whose absorption properties are well established. The window material employed in the LNFC is silicon nitride. In general, the LNFC is chemically compatible with water and iso­propyl alcohol; however, it is not compatible with acetone.

(ii) Xenocs Couette stage for shear SAXS: in-air operated coaxial cylindrical shear stage with an external cylinder rotor (Couette type) and temperature control of the stator. It operates with a 1 mm fixed gap between the stator and internal surface of the rotor, and supports rotational and oscillatory modes.

(iii) Advanced GISAXS module for thin films including Omega, Phi and Psi rotations.

(iv) Multi-sample holder specifically designed for powders and gels (also suitable for liquids).

(v) Multi-purpose X-ray temperature stage (−20 to 150°C): Peltier-type temperature stage for heating and cooling adapted for solids, gels, powders and capillaries. Compatible with GISAXS and the Xenocs LNFC temperature range reduced to 4–50°C for liquid samples.

## 3. Instrument performance

We describe in this section the performance of the Xeuss 3.0 HR instrument installed at the BioSAXS facility at ITACA.SB.

### 3.1. BioSAXS and SEC-SAXS on proteins

SAXS data were collected in vacuum using the Ga metal-jet microsource and the EIGER 2R 1M detector, changing the SDD from 257.4 to 1800 mm, depending on the expected size of the proteins. The following proteins were selected as case studies: lysozyme from chicken egg white (L6876-10G, 14 kDa), recombinant human insulin (I2643-25MG, 35 kDa), carbonic anhydrase II (C2522-5MG, 29 kDa), bovine serum albumin (monomer 66 kDa and dimer 133 kDa forms, A7030-10G) and apoferritin from horse spleen (A3641-100MG, 476 kDa). All samples were prepared in SAXS-compatible buffers optimized for each protein, to ensure monodispersity and protein stability, and to minimize potential aggregates. Weighted amounts of lyophilized proteins were dissolved in the corresponding buffers to achieve the desired final concentration. All solutions were filtered using 0.22 µm cellulose acetate filters prior to measurement.

In batch SAXS experiments, purified samples are directly loaded into the exposure cell and measured at a defined concentration. Experimental parameters, namely SDD, beam collimation and exposure time (see Table 1 ▸), were preliminarily optimized to achieve the best compromise between accessible *q*-range, signal-to-noise ratio, detector saturation and informative content of the SAXS profile. In Section S1 of the supporting information we provide an example of this preliminary test for apoferritin and insulin.

In SEC-SAXS, the sample is first injected in a size-exclusion column (Cytiva Superdex 200 Increase 10/300 GL) equilibrated in the measurement buffer (100–200 µl injected volumes at 0.5 ml min^−1^). Upon detection of the eluting protein peak, the flow rate is manually reduced (0.1 ml min^−1^) to ensure sufficient exposure time per frame. Scattering data are then continuously collected across the chromatographic peak (30 s exposures). The protein concentration in the X-ray-exposed volume is monitored in real time by in-line UV absorbance at 280 nm (see Fig. S3 of the supporting information), allowing frame selection corresponding to the monodisperse species. While batch SAXS is suitable for stable and homogeneous samples, SEC-SAXS enables in-line separation of aggregates or oligomeric mixtures immediately prior to exposure, improving data quality for challenging systems (Bucciarelli *et al.*, 2018 ▸). In all cases, SAXS data were pre-processed using *XSACT 2.10* (Xenocs, 2023 ▸); SEC-SAXS datasets were further processed in *BioXTAS RAW* (Hopkins *et al.*, 2017 ▸), which was used for peak frame selection, buffer frame selection and buffer subtraction (see Fig. S4 of the supporting information). The scattering curves were obtained by subtracting the buffer contribution from the sample signal 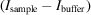, resulting in data on a relative scale. Subsequently, SAXS data were analyzed with the program *ATSAS* (version 4.1.1) (Franke *et al.*, 2025 ▸), a program suite for small-angle scattering data analysis from biological macromolecules, by computing the Guinier plot, pair distance distribution, gyration radius and molecular weight (Table 1 ▸). The atomic models associated with the corresponding Small Angle Scattering Biological Data Bank (SASBDB) entries (https://www.sasbdb.org/; Kikhney *et al.*, 2020 ▸), as reported in Table 1 ▸, were downloaded and compared with the low-resolution shapes obtained from SAXS data.

#### 3.1.1. Main requirements – batch SAXS or SEC-SAXS on proteins

The main requirements for performing batch SAXS and SEC-SAXS measurements on proteins at the BioSAXS facility at ITACA.SB, focusing on the experimental conditions used in this work, are listed in Section S2 of the supporting information and summarized here in Table 2 ▸.

Batch SAXS measurements are suitable for well behaved, monodisperse samples, whereas SEC-SAXS is employed when in-line purification is required to separate coexisting species or to mitigate sample heterogeneity through in-line size-exclusion chromatography. However, batch SAXS can also be applied to non-monodisperse systems, for example to assess sample heterogeneity or to investigate concentration-dependent equilibria (*e.g.* monomer–dimer transitions), depending on the specific experimental objective.

#### 3.1.2. Experimental considerations for laboratory SAXS

In addition to sample-related requirements, specific experimental conditions must be considered when using laboratory-based SAXS instruments. Radiation damage can affect data quality, particularly due to the longer exposure times required compared with synchrotron measurements. In our setup, this effect is mitigated by optimizing exposure times and, in SEC-SAXS mode, by maintaining continuous sample flow to ensure constant renewal of the irradiated volume. Radiation damage was systematically monitored by comparing consecutive frames, and no significant radiation-induced changes were observed under the exposure conditions employed in this study. For SEC-SAXS measurements, the flow rate represents an additional critical parameter. In our experiments, a flow rate of 0.1 ml min^−1^ was used as a compromise between chromatographic resolution, sample dilution and sufficient exposure time in the X-ray beam, which is particularly important for laboratory-based sources.

### 3.2. SAXS on micelles

SAXS data were collected on PS20 (Polysorbate 20 or Tween 20) and VitE-TPGS (vitamin E–d-α-tocopherol polyethyl­ene glycol 1000 succinate) using the Ga metal-jet microsource and the EIGER 2R 1M. Samples were loaded into a 2 mm BioCube capillary for measurements. For the PS20 solution (5 mg ml^−1^), data were acquired at two different configurations: first, at an SDD of 600 mm utilizing a 1.3 × 1.3 mm beam with a total integration time of 1 h (60 frames at 60 s each); second, at an SDD of 1800 mm with a 0.5 × 0.5 mm beam for a total of 10 h (60 frames at 600 s each). Similarly, VitE-TPGS (0.4 wt%) was characterized at both distances. At SDD = 600 mm setting (1.3 × 1.3 mm beam), the sample was exposed for 1 h (60 frames at 60 s each). For the 1800 mm configuration (0.5 × 0.5 mm beam), the total exposure time was 5 h (60 frames at 300 s each).

The *XSACT* software was employed for the automated calibration and azimuthal integration of scattering patterns into 1D profiles, facilitating the simultaneous extraction and visualization of all frames. The scattering curves were converted to an absolute scale using water normalization, according to 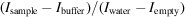 × 0.0164 cm^−1^.

**Figure 4:**
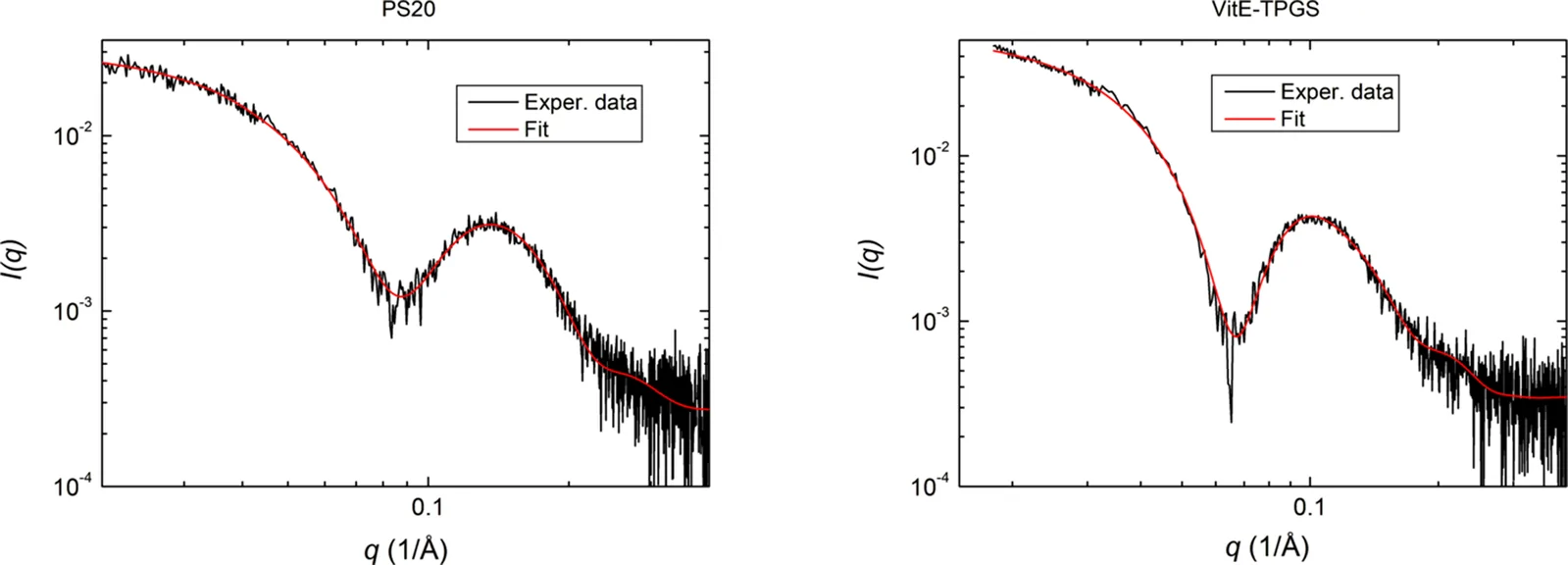
SAXS data and relative fits for the PS20 (left panel) and VitE-TPGS (right panel) micelles.

SAXS data were further analyzed by a novel approach, based on the use of the Nyquist–Shannon theory, recently developed by De Caro *et al.* (2026 ▸). Fig. 4 ▸ shows the experimental and fitted profiles for the PS20 and VitE-TPGS micelles. The structural parameters derived from the fits are summarized in Table 3 ▸.

### 3.3. Scanning SWAXS microscopies

The selected samples for SWAXS microscopy include systems with markedly different densities, namely peptide-based fibers and bone tissues, highlighting how experimental parameters such as sample thickness, beam size, scanning step and exposure time were adapted according to the sample characteristics and the required spatial resolution. In particular, the denser bone specimens were prepared with a thickness approximately one order of magnitude less than that of the peptide-based fibers in order to achieve a comparable signal-to-noise ratio within reasonable acquisition times at each sample position. The reported fields of view (FOVs) are sample dependent and were selected according to the investigated regions of interest.

All 2D SAXS and WAXS data were automatically calibrated, as described in Section 2, and azimuthally integrated by the software *XSACT* into 1D profiles. Additionally, scanning SWAXS microscopy data were processed through the proprietary *SUNBIM 4.0* software (Scattarella *et al.*, 2025 ▸).

Table 4 ▸ summarizes the main experimental configurations used for the measurements reported in this section.

#### 3.3.1. Peptide-based fibers

Structural characterization of peptide-based fibers was performed on the (F-Dopa)3 peptide fibers (Diaferia *et al.*, 2020 ▸). The molecular components of the fibers are as follows:

(i) l-Phenyl­alanine (F): a highly hydro­phobic aromatic amino acid which serves as the primary ‘structural engine’, driving the self-assembly process through strong π–π stacking and hydro­phobic interactions. It is essential for the formation of stable, ordered architectures such as nanofibers or nanotubes.

(ii) l-Dopa (Dopa): a catechol-containing amino acid inspired by the ‘mussel adhesive proteins’. The presence of two hydroxyl (–OH) groups on the aromatic ring confers unique properties on the peptide, including exceptional surface adhesion, redox activity and the ability to coordinate metal ions.

**Figure 5:**
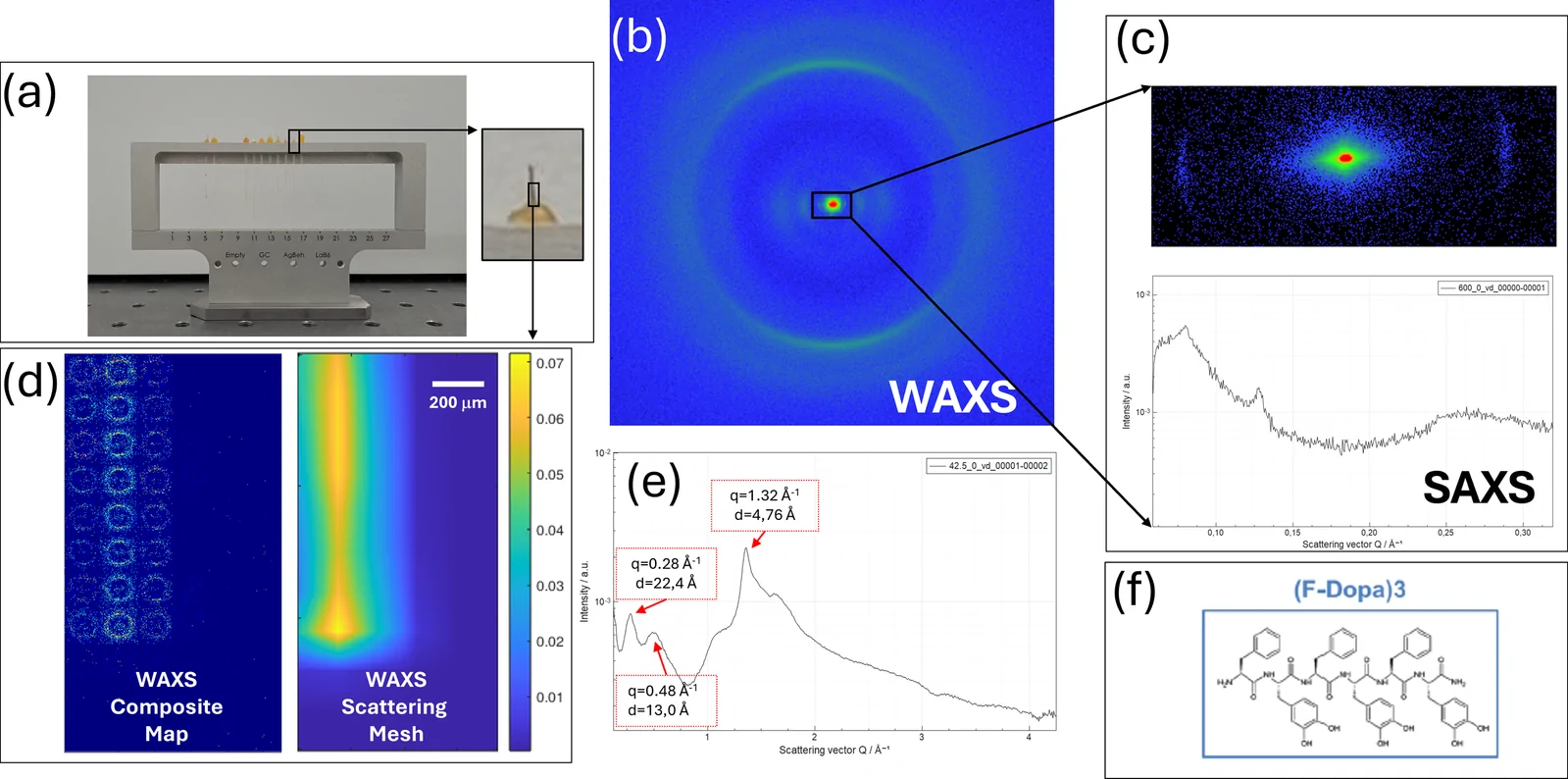
Scanning WAXS data of peptide-based fiber. (*a*) View of the multi-sample holder utilized for the analysis of the fibrous specimens; the inset provides a close-up of a single fiber mounted for measurement. (*b*) Representative 2D WAXS pattern showing the characteristic scattering rings used for phase identification and structural analysis. (*c*) Detail of the central SAXS region and the corresponding 1D SAXS profile (intensity versus *q*), illustrating the scattering features at low angles. (*d*) Scanning microscopy results: the WAXS composite map and the corresponding WAXS scattering mesh, obtained after an interpolation procedure (Siliqi *et al.*, 2016 ▸), demonstrate the spatial distribution and intensity variations of the crystalline signal across the sample surface (dimensions in mm). (*e*) Representative 1D WAXS profile integrated from the 2D data, showing the meridional and equatorial peaks used for the structural fingerprinting of the fiber. (*f*) Structural representation of (F-Dopa)3 peptide.

SWAXS data were collected using the Ga metal-jet microsource, with a beam aperture of 150 µm, at two distinct SDDs: 600 mm (SAXS) and 42.5 mm (WAXS). Each single-point acquisition was performed with an exposure time of 1800 s utilizing the detector in line eraser (LE) mode which intrinsically requires two acquisitions at shifted detector positions (1800 s × 2 = 3600 s total). The two patterns are then merged into a composite image to eliminate the detector gap. In addition, scanning X-ray microscopy was performed at both distances over an area of 0.7 × 1.5 mm to evaluate the lateral structural homogeneity of the fiber. The maps were acquired with a spatial resolution of 150 µm (Δ*x* = Δ*z*) and a shortened exposure time of 30 s per point in LE mode (total exposure time 30 × 2 = 60 s per point). This multi-distance scanning approach allowed for the simultaneous mapping of the crystalline stacking of phenyl­alanine/DOPA residues and the nanoscale fibrillar morphology. Fig. 5 ▸ shows the WAXS and SAXS data, integrated across the fibers, and the WAXS scanning microscopy maps (composite and scattering) displaying the spatial intensity distribution of the crystalline phase. The WAXS data correspond to the expected cross-β fiber diffraction 2D pattern (Diaferia *et al.*, 2020 ▸); in particular, the pattern presents one peak along the meridional direction (*i.e.* the fiber axis), around 4.8 Å, which corresponds to the β-strand distance, and another along the equatorial direction (*d* range 13–22 Å), which corresponds to the inter-sheet distance. The SAXS data indicate a clear additional nanoscale periodicity.

#### 3.3.2. Bone tissues

**Figure 6:**
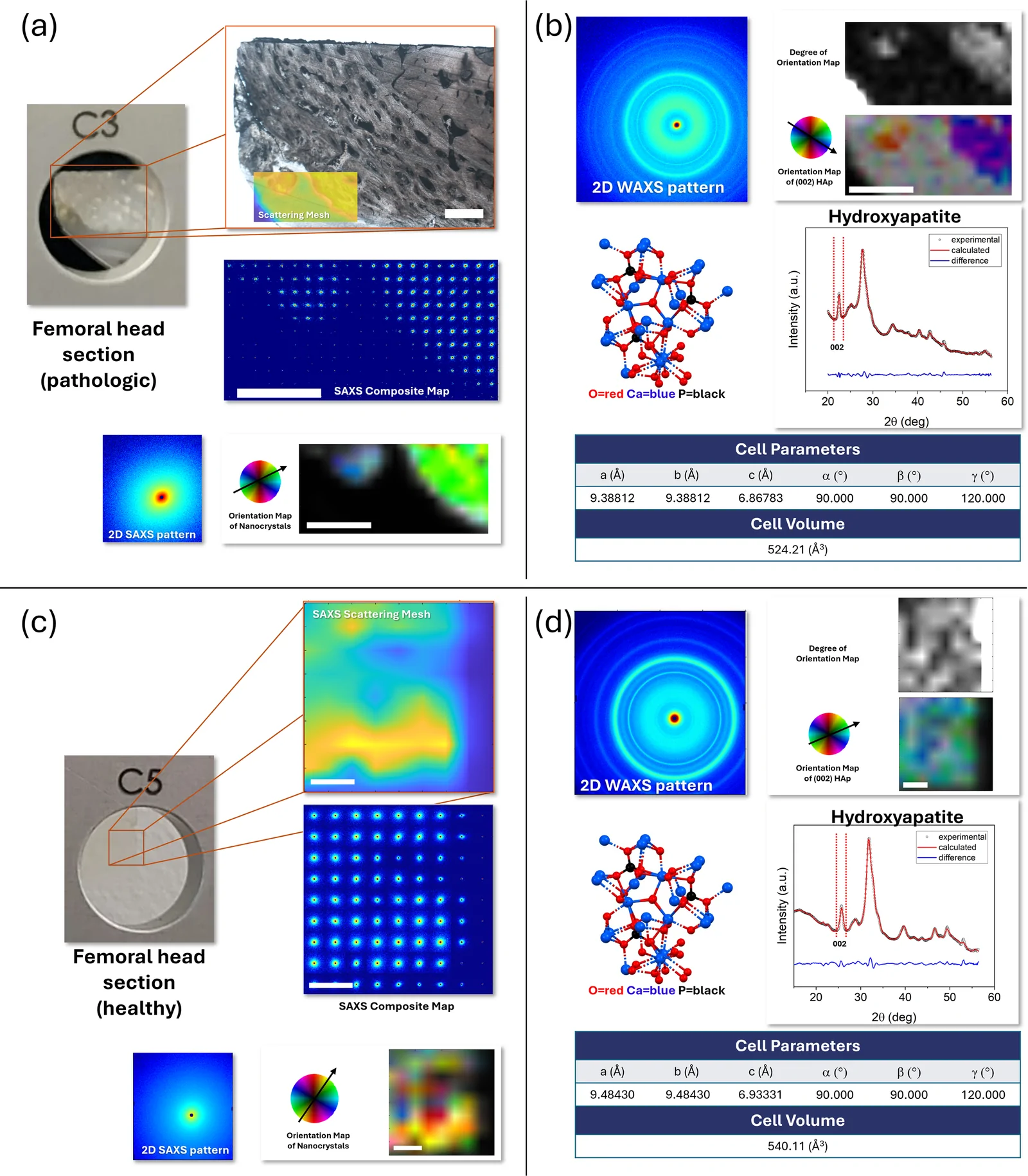
Scanning SWAXS data of bone biopsies. (*a*–*b*) Pathological femoral head core biopsy (dwarfism-affected sample). (*a*) Macroscopic view of the specimen mounted on the sample holder together with the corresponding optical micrograph showing the scanned area; below, the averaged 2D SAXS pattern over the entire scanned region, the composite SAXS map illustrating the spatial distribution of the scattering intensity across a 1 mm scale, and the resulting orientation map of the collagen nanocrystals are shown. (*b*) Sub-molecular analysis of the mineral phase: averaged 2D WAXS pattern over the scanned area displaying the characteristic diffraction rings of HAp. The degree of orientation and orientation maps of the 002 HAp reflection highlight the local mineral organization. The 1D integrated WAXS profile (experimental data in black) is shown with the corresponding Rietveld refinement fit (calculated model in red, difference plot in blue), where the 002 peak is specifically marked. The crystal structure of HAp (O = red, Ca = blue, P = black) and a table summarizing the refined unit cell parameters and cell volume are also provided. (*c*–*d*) Healthy femoral head biopsy: the same set of analyses is shown, including averaged SAXS and WAXS patterns over the scanned region, SAXS composite map, SWAXS-derived orientation maps and 1D WAXS profile with Rietveld refinement, as described above. White scale bars correspond to 1 mm.

Structural scanning SAXS/WAXS microscopy was performed on bone tissue biopsies to map above-molecular (SAXS) and sub-molecular (WAXS) heterogeneities within the collagen Type I and hy­droxy­apatite (HAp) matrix. Pathological (affected by dwarfism) and healthy femoral neck specimens were retrieved during surgery and fixed in 4% buffered formalin. Each slice was sectioned to a thickness of 100 ± 20 µm and embedded in polymethyl­methacrylate (PMMA) for handling and analysis. Written informed consent was obtained from all patients, following the ethical protocols of the Rizzoli Orthopedic Institute in compliance with Italian and European regulations (Giannini *et al.*, 2012 ▸). The specimens were mounted on a standard multi-sample holder for solids [pathological: C3 in Fig. 6 ▸(*a*); healthy: C5 in Fig. 6 ▸(*b*)]. Scanning areas of 3 × 3 mm and 4 × 4 mm were covered for the pathological and healthy specimens, respectively, for both techniques.

The dwarfism-affected specimen was measured using the Ga metal-jet microsource with a beam aperture of 150 µm. The SDD was set at 42.5 mm for WAXS and 1800 mm for SAXS. Data were acquired with an exposure time of 30 s per point and a scanning step of 150 µm, utilizing the detector in LE mode (30 s × 2 = 60 s total exposure time per point).

The healthy specimen was measured using the Cu GENIX^3D^ microfocus source with a scanning step of 500 µm and an exposure time of 60 s per point in LE mode (60 s × 2 = 120 s total exposure time per point).

Data reduction and analysis were performed using the *XSACT* and *SUNBIM 4.0* software packages; in particular, individual diffraction patterns were pre-processed in *XSACT*, while the final microscopy mapping was generated via *SUNBIM*.

Fig. 6 ▸ compares scanning SWAXS results from pathological and healthy femoral biopsies. For both samples [panels (*a*) and (*c*)], the composite SAXS map is shown together with the 2D SAXS frame integrated across the entire scanned area. The orientational map for the collagen fibers was generated by point-by-point integration of the first meridional SAXS reflection at Δ*q* = 0.0090–0.0119 Å^−1^ (52.9–69.6 nm). Similarly [panels (*b*) and (*d*)], the 2D WAXS patterns were integrated over the full scanned region to produce a single 1D profile for each sample, which was identified as the hy­droxy­apatite (HAp) crystal phase. A Rietveld refinement was subsequently performed on both profiles; the resulting fits and refined lattice parameters are presented in the figure. In both cases, 2D WAXS microscopy maps, representing both the degree and direction of orientation, were produced by mapping the 002 HAp reflection in the range Δ*q* = 1.8051–1.9061 Å^−1^ (0.330–348 nm), as indicated by the vertical dashed bars in the Rietveld plots.

### 3.4. USAXS/SAXS/WAXS

**Figure 7:**
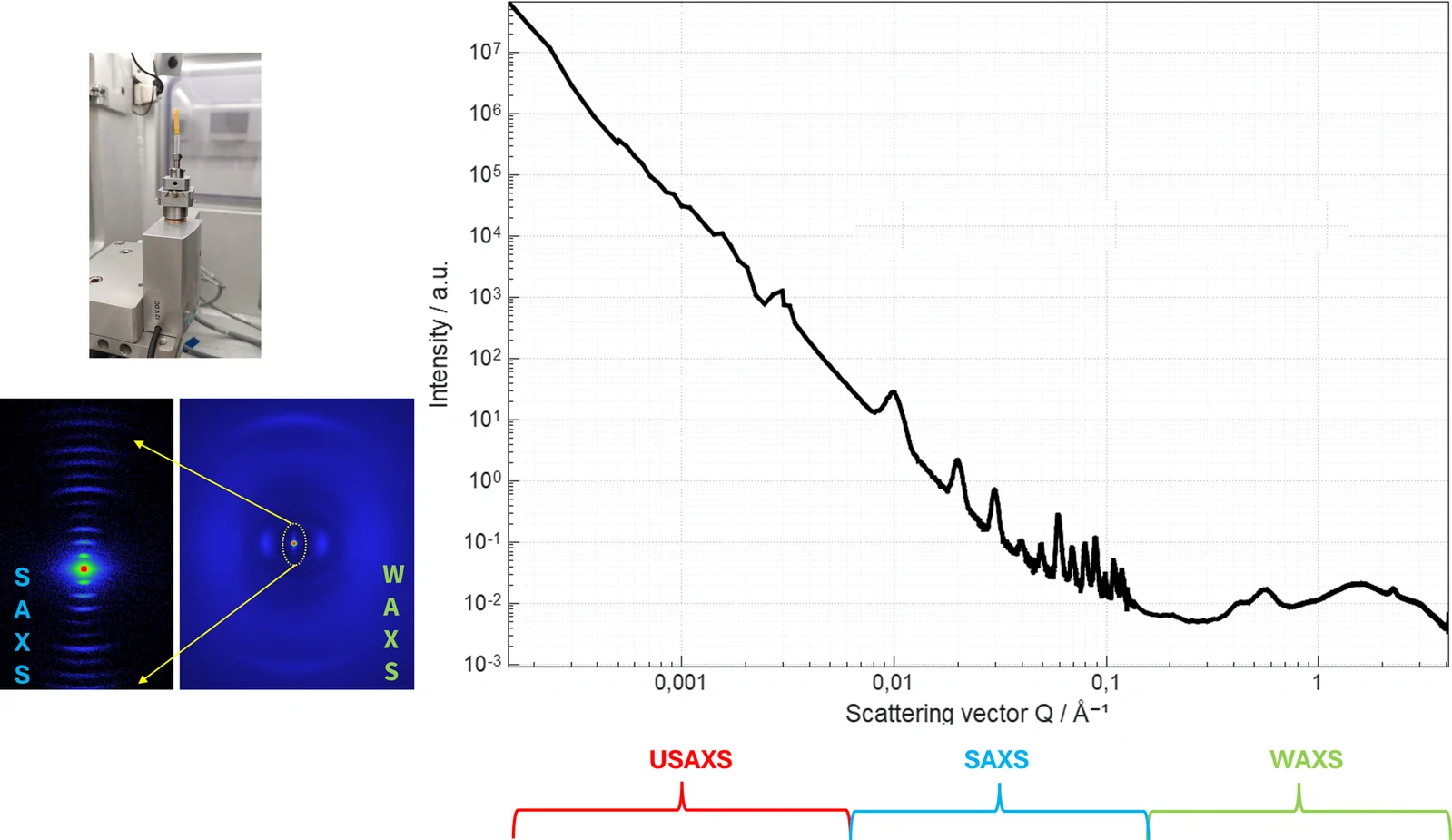
Structural characterization via USAXS/SAXS/WAXS was performed on core biopsies harvested from the deep digital flexor tendon of a 4 year-old male *Equus caballus* (EQ). The specimens were encapsulated within 3 mm-diameter Kapton capillaries and positioned on a motorized rotary stage for analysis (refer to the top-left inset).

In this experiment, USAXS, SAXS and WAXS data were collected on core biopsies excised from the deep digital flexor tendon of a 4 year-old male *Equus caballus* (EQ), inserted in a Kapton capillary of 3 mm diameter and mounted on a rotary stage (see Fig. 7 ▸). Despite the absence of expected structural features in the USAXS region for this specific example, data were acquired across this extended range to showcase the comprehensive multi-scale analysis capabilities of the equipment. We worked with the Ga metal-jet microsource (as already mentioned in Section 2), utilizing a beam size of 3 × 2.5 mm for USAXS and 0.5 × 0.5 mm for SAXS/WAXS. For both USAXS and SAXS/WAXS measurements, data were acquired on two points: one within the sample and another in a no-sample region to evaluate the background signal from the Kapton and subtract it from the sample signal. Collection times were 15 min per scan of the analyzer rocking curve for USAXS and 120 s per point for SAXS/WAXS data. USAXS data reduction, performed using *XSACT*, consists of two dedicated modules: subtraction followed by desmearing. The USAXS subtraction module is simply used to subtract two sets of USAXS data. It includes automatic normalization and relative *q* shift, if required. The *q* shift is implemented in order to correct for zero angle errors in the scans, as a result of any mechanical and thermal instabilities. The *q* shift is applied prior to the subtraction. The subtracted USAXS data can be entered into the desmearing module. The desmearing is done via the well established Lake algorithm (Lake, 1967 ▸) which is used on 1D data acquired with a finite-size beam, aiming to retrieve the original scattering profile from smeared data. Extrapolation of data is completed to recover original slopes. USAXS, SAXS and WAXS data are shown in Fig. 7 ▸, covering the range from *q* = 0.000152 Å^−1^(*d* ∼ 4.1 µm, USAXS) to *q* = 4.103970 Å^−1^ (*d* ∼ 1.5 Å, WAXS).

## 4. Perspectives

The results presented here demonstrate the ability to perform scanning SWAXS microscopy with micrometre resolution, opening new avenues for materiomics (Cranford *et al.*, 2013 ▸), and allowing for the high-throughput screening of both natural and synthetic hierarchical materials. Future perspectives include the integration of *in situ* mechanical testing or microfluidic environments to monitor structural rearrangements in real time, providing a deeper understanding of the structure–function relationship in complex tissues and advanced functional materials.

Regarding biological macromolecules, the integration of SEC-SAXS with high-brilliance laboratory sources marks a significant step towards routine, high-resolution structural biology. The aim is to automate these pipelines to characterize protein–protein interactions and large macromolecular complexes under near-physiological conditions. Expanding the detector capabilities will further enable the simultaneous tracking of folding/unfolding dynamics, bridging the gap between static crystal structures and the functional flexibility of proteins in solution.

Finally, from a clinical standpoint, the versatility of this scanning microdiffraction approach enables the investigation of structural markers associated with a broad range of pathological conditions directly within biopsy specimens. This methodology facilitates the identification of early ‘structural fingerprints’ and hierarchical molecular alterations that precede macroscopic clinical manifestations. Such an approach opens new avenues for enhancing diagnostic precision and monitoring therapeutic efficacy by analyzing fundamental structural changes within biological tissues, regardless of their specific composition or nature.

The laboratory is part of a the strategic national infrastructure ITACA.SB and provides access to state-of-the-art SWAXS methodologies and SEC-SAXS pipelines with automated sample handling, high-throughput data collection and real-time data analysis. Access to the facility is open to both national and international users (https://www.itaca-sb.it/tna/), fostering collaborative projects across biology, materials science and biomedical research.

## Supplementary Material

Supporting information. DOI: 10.1107/S1600576726007132/uz5036sup1.pdf

## Figures and Tables

**Table 1:** Macromolecular properties extracted from SAXS and SEC-SAXS data The experimental technique used for each dataset (batch SAXS or SEC-SAXS) is explicitly indicated in the second column, together with main experimental parameters: SDD, exposure time per frame and beam collimation. Comparative references to the SASBDB codes are provided, together with the expected molecular weight (MW_exp_) and radius of gyration (Rg_exp_) to validate the in-house measurements against structural data. SAXS profiles were analyzed by *ATSAS 4.1*, including *DAMMIFab initio* modeling. The *ATSAS* package allows determination of the Rg value (here Rg_data_) and different methods for the MW evaluation. Here, we report the evaluated MW_data_ along with its probability. In the ‘Raw Data’ column, black circles represent the experimental scattering data, while the red solid lines correspond to the fitted scattering curves.

[Chem scheme1] 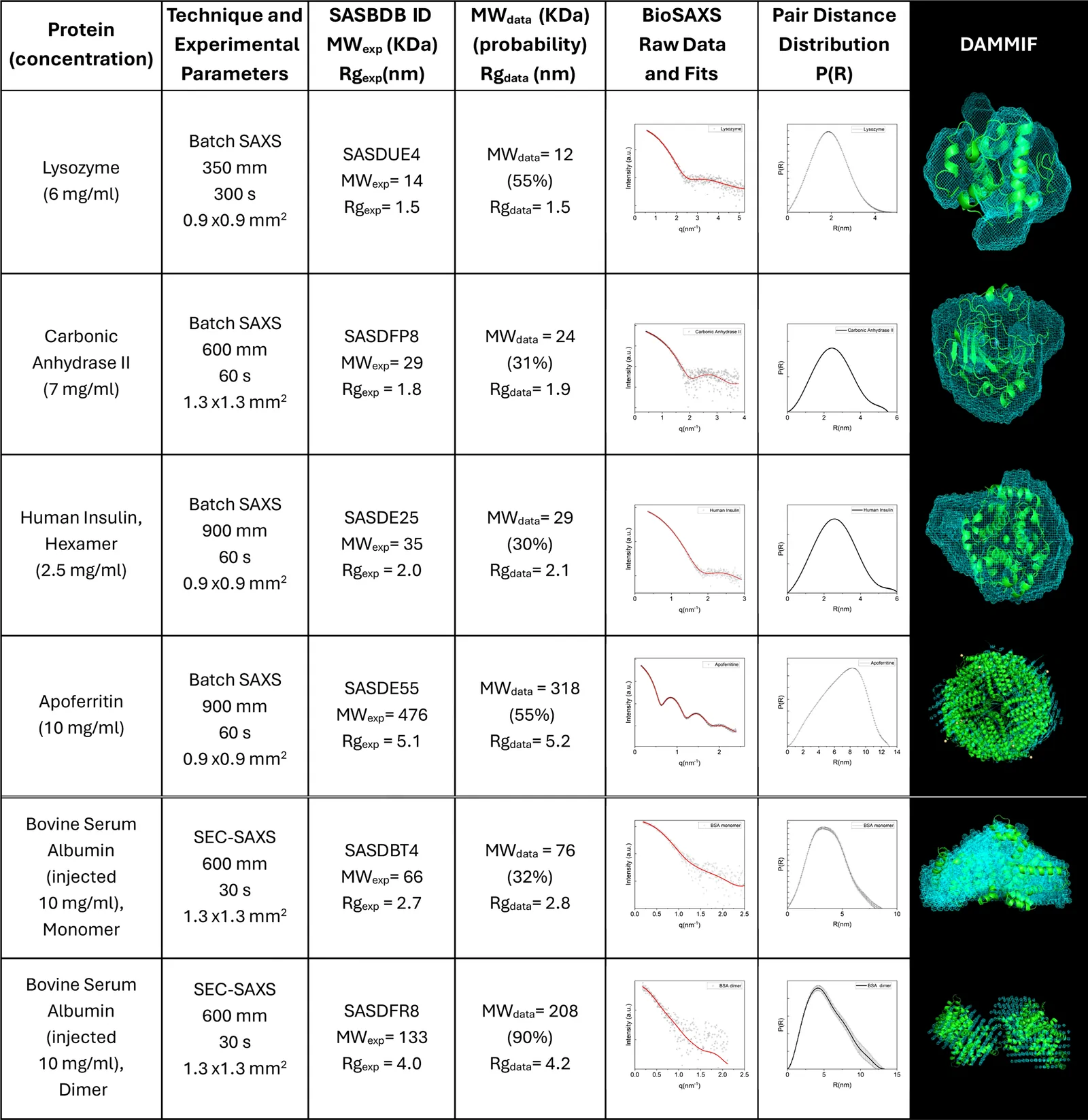

**Table 2:** Practical requirements and experimental guidelines for batch SAXS and SEC-SAXS measurements on macromolecules at the BioSAXS laboratory of the research infrastructure ITACA.SB The criteria reflect both sample quality requirements and key experimental conditions specific to the laboratory-based SAXS setup.

Parameter	Requirement (batch SAXS)	Requirement (SEC-SAXS)
Purity	≥90–95%	≥90–95%
Monodispersity	Single oligomeric species, no aggregates	SEC separates species in-line
Concentration	≥3 concentrations (*e.g.* 1–10 mg ml^−1^)	Highest feasible concentration (5–10 mg ml^−1^ typical)
Volume required	∼50–100 µl total stock (15 µl for single measurement)	100–200 µl injection volume
Buffer matching	Identical buffer for background subtraction	Column-equilibrated buffer
Buffer compositions	Salt < 1 *M*, glycerol ≤ 10–30%, minimal additives	Same constraints; avoid excess detergent
Detergents	Below critical micelle concentration	SEC strongly recommended if present
Sample preparation	Filtered/centrifuged before measurement	Filtered/centrifuged prior to injection
Stability	Stable during measurement; no precipitation	Stable throughout chromatography run
Flow rate		0.1 ml min^−1^ (optimized for laboratory-source SAXS)
Radiation damage control	Exposure optimization and monitored via frame comparison	Minimized by continuous flow ensuring sample renewal and monitored via frame comparison

**Table 3:** Summary of the micelle’s shape parameters obtained from PS20 and VitE-TPGS laboratory data 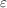
 = core ellipticity; 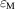 = micelle ellipticity; 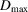 = micelle’s maximum size; 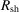 = shell thickness; 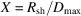; 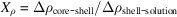, where the 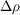 are the electron density contrasts.

	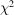	ɛ	ɛ_M_	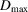 (Å)	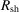 (Å)	*X*	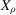
PS20	1.05	1.75 ± 0.01	1.39 ± 0.02	94.5 ± 0.5	16.06 ± 0.5	0.170 ± 0.006	−2.20 ± 0.05
VitE-TPGS	1.20	1.63 ± 0.01	1.36 ± 0.02	121.5 ± 0.5	18.8 ± 0.5	0.155 ± 0.005	−1.75 ± 0.05

**Table 4:** Summary of the experimental configurations used in the scanning SWAXS measurements (parameters are detailed in the text)

Sample	Thickness (mm)	Exposure time per step (s)	Beam size (mm)	Scanning step (mm)	FOV (mm)	Source
Peptide-based fiber	∼1	60	0.15 × 0.15	0.15	0.75 × 1.5	Ga metal-jet
Bone tissue (pathologic)	∼0.1	60	0.15 × 0.15	0.15	3 × 3	Ga metal-jet
Bone tissue (healthy)	∼0.1	120	0.5 × 0.5	0.5	4 × 4	Cu GENIX^3D^
