# Supplementary material for: The BioSAXS laboratory of the ITACA.SB Research Infrastructure
Source: J Appl Crystallogr. 2026 Aug 24;59(Pt 5):1757–68. doi: 10.1107/S1600576726007132 (PMC13636039; doi:10.1107/S1600576726007132)
Supplement: Supplementary file 1 [file j-59-01757-sup1.pdf]

JOURNAL OF
APPLIED
CRYSTALLOGRAPHY

Volume 59 (2026)

Supporting information for article:

The BioSAXS laboratory of the ITACA.SB Research Infrastructure

Francesco Scattarella, Davide Altamura, Vincenzo Mangini, Teresa Sibillano, Erika Manicone, Rocco Lassandro, Liberato De Caro, Gemma Newby and Cinzia Giannini

S1. SAXS profiles acquired at different sample-to-detector distances and beam collimations

This section reports the SAXS profiles of apoferritin (Figure S1) and insulin (Figure S2) acquired using different combinations of sample-to-detector distance (SDD) and beam collimation (see Table S1). During the measurements, the experimental configuration was selected following a practical optimization workflow aimed at maximizing the accessible q-range and informative content of the scattering profile, while preserving data quality. However, since the experimental setup operates without a beamstop, the beam size and intensity were carefully controlled to avoid saturation of the detector by the transmitted beam. It is important to emphasize that the scattering profiles acquired at different detector distances were intentionally not merged. Indeed, they have different statistics which affects the intensity errors, so a brutal merging procedure might introduce additional uncertainties and degrade rather than improve the overall data quality. As a consequence, we selected the most informative profiles (shown in the main text) to be analysed.

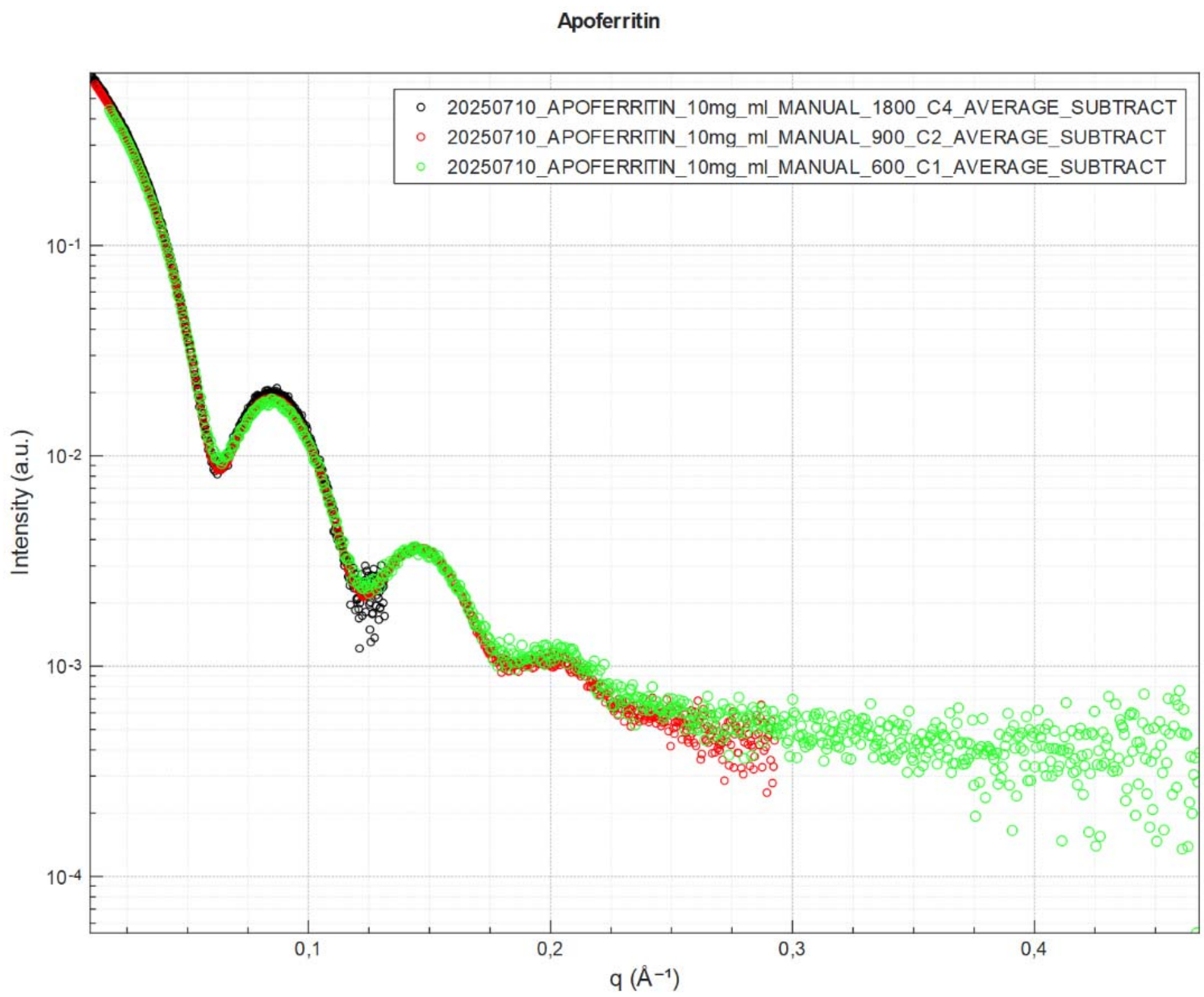

Figure S1. SAXS scattering profiles of apoferritin acquired at three sample-to-detector distances (600, 900, and 1800 mm), each measured using the corresponding optimized incident beam collimation.

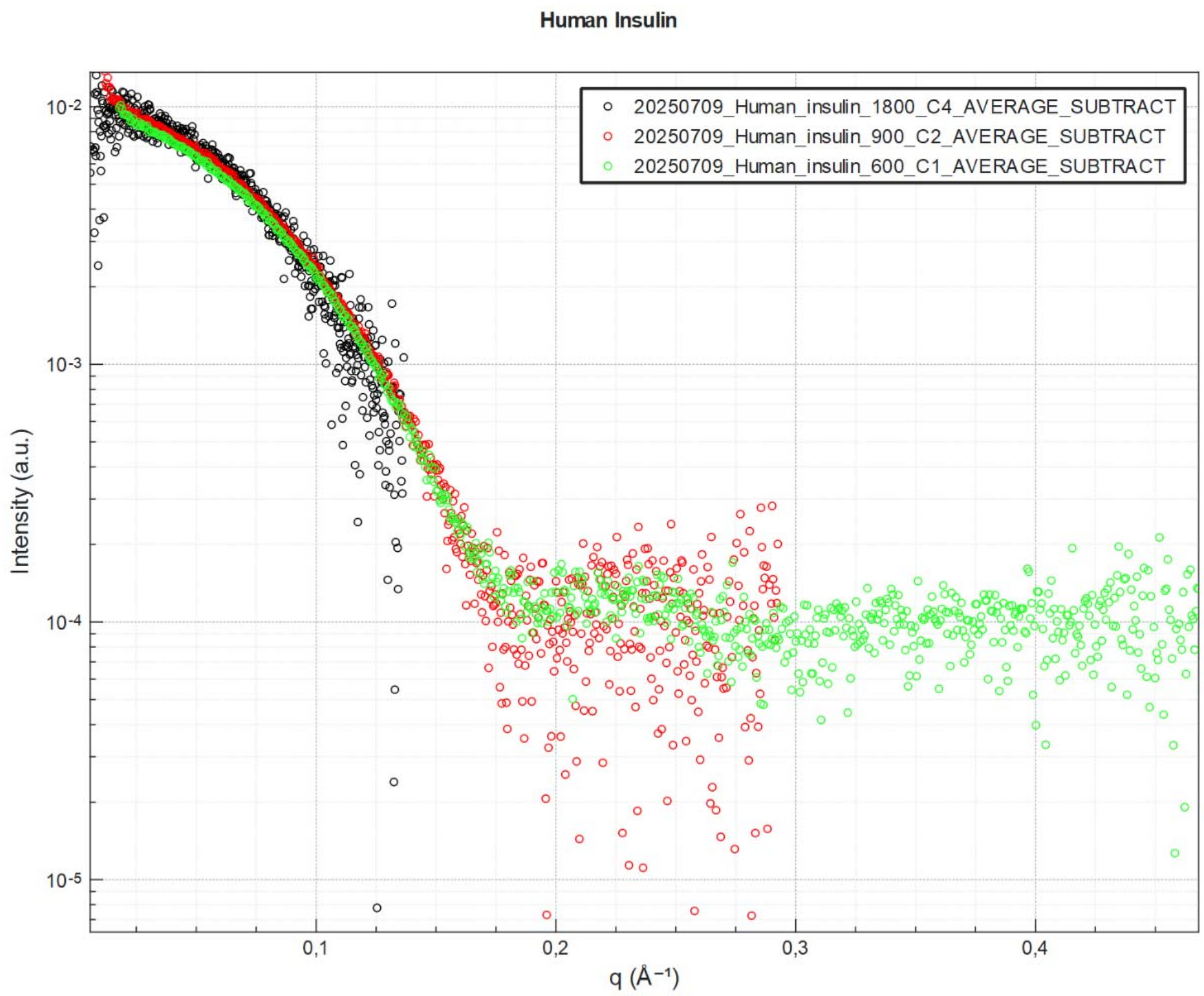

Figure S2. SAXS scattering profiles of apoferritin acquired at three sample-to-detector distances (600, 900, and 1800 mm), each measured using the corresponding optimized incident beam collimation.

SDD (mm)	Collimation (mm^2)	*q*-range ($Å^{-1}$)
1800	C4 = 0.5x0.5	~0.0099÷ 0.1314
900	C2 = 0.9x0.9	~0.0119÷ 0.2925
600	C1 = 1.3x1.3	~0.0179÷ 0.4681

Table S1. Experimental configurations used for the SAXS measurements reported in this work for apoferritin and insulin. For each acquisition, the sample-to-detector distance, incident beam collimation and accessible *q*-range are listed. The combinations of detector distance and beam collimation were selected to optimize the balance between low-*q* accessibility and signal-to-noise ratio.

S2. Batch SAXS or SEC-SAXS on proteins: sample requirements

High sample quality is essential for obtaining reliable SAXS data. In particular, aggregation or polydispersity significantly compromises data quality. Therefore, protein samples should meet the following criteria.

Purity and monodispersity

Aggregation or polydispersity significantly compromises SAXS data quality. Therefore, specimens should guarantee:

- High purity (ideally ≥ 90-95%)
- For batch SAXS the sample must be monodisperse, containing a single oligomeric species

- No detectable aggregates or precipitates

Concentration Series (Batch SAXS measurements)

Measuring multiple concentrations allows the detection of interparticle effects (repulsion or attraction) and the identification of aggregation at higher concentrations. It is advisable to:

- Provide at least three concentrations (e.g. 1, 5, 10 mg/ml) or a sufficient amount of the sample to prepare the desired concentrations prior to measurements
- Concentration must be accurately determined
- Samples should be centrifugated (e.g. 10-15 min, 5000 g, 4°C) or filtered (0.02 µm) prior to measurement

Sample volume and quantity

Here are quantities for batch SAXS and SEC-SAXS mode.

For Batch SAXS

- Typical measurement volume: 10-50 µl (depending on setup)
- Recommended to provide 100 µl of stock solution to allow preparation of dilution series
- Use the highest feasible concentration (often 5-10 mg/ml)

For SEC-SAXS

- Injection volume: typically 100-200 µl
- Use the highest feasible concentration (often 5-10 mg/ml)
- Provide sufficient buffer (500 ml or 1 l) for column equilibration and running

SEC-SAXS is recommended for oligomerization prone samples, polydisperse samples, membrane proteins, sample containing detergents

Buffer requirements

Precise buffer matching is essential for accurate background subtraction.

- The buffer used for background subtraction must be identical to that of the proteins sample (same salts, pH, additives, reducing agents, etc)
- Buffer must be measured separately before and/or after the sample

Buffer composition

Excess additives increase background scattering and reduce signal quality

- Salt concentration: preferably < 1M
- Glycerol: ideally 5-10%, no more 30%
- Small ligands (ATP, ADP cofactors): typically < 10 mM
- Detergents (if required): minimal concentrations, avoid micelle formation (< critical micelle concentrations, CCMs)
- For membrane proteins, SEC-SAXS is strongly recommended

Sample stability

- The protein must be stable over the duration of the experiment
- No time-dependent aggregation or degradation
- Provide storage conditions (4°C, frozen, additives etc.)
- Avoid freeze-thaw cycles if possible
- If radiation sensitivity is expected, this should be indicated

Additional information

For optimal experiment planning, provide the following information:

- Amino acid sequence
- Molecular weight
- Extinction coefficient
- Oligomeric state (if known)
- Cofactors or ligands required for stability/activity
- Previous quality control data

S3. SEC chromatogram for BSA

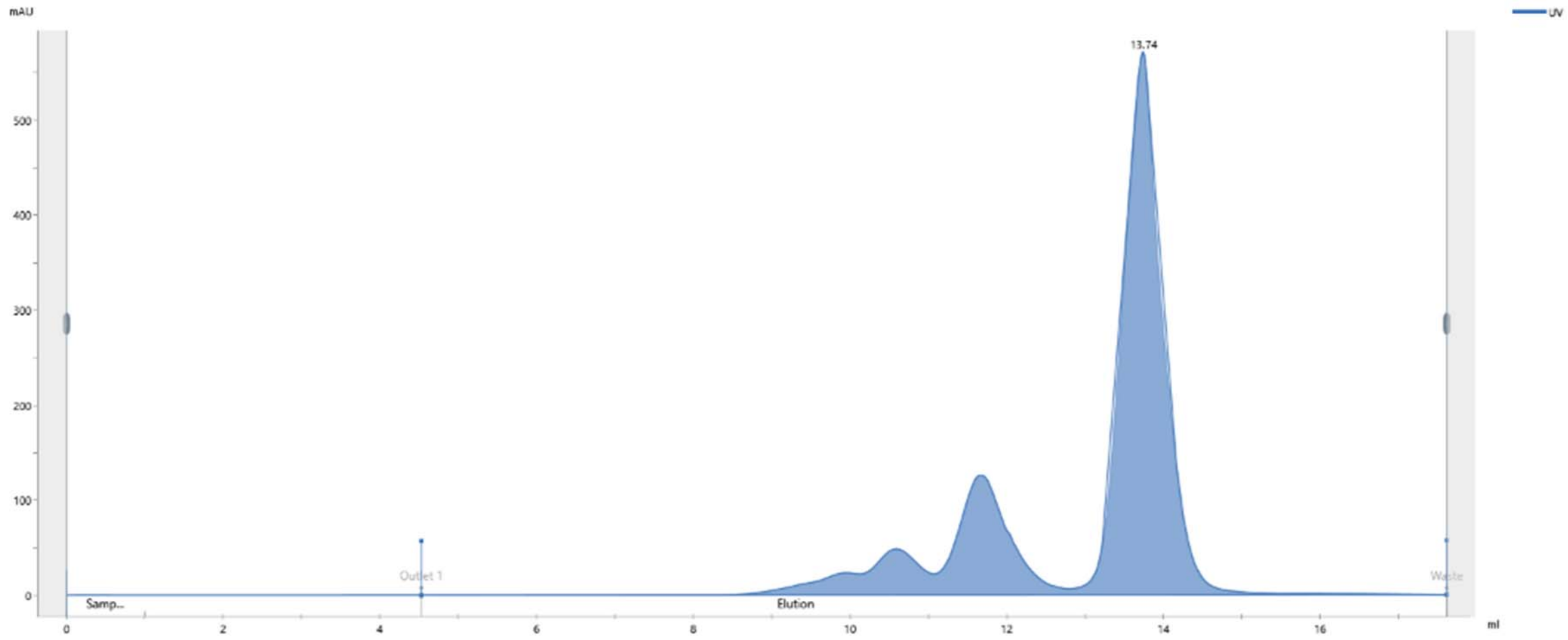

Figure S3. UV absorbance (mAU) chromatogram of BSA (10 mg/mL) versus elution volume, collected during SEC-SAXS. The profile illustrates the separation of species during the chromatographic run, with the main peak corresponding to the predominant monomeric form and earlier elution features indicating higher-molecular-weight species.

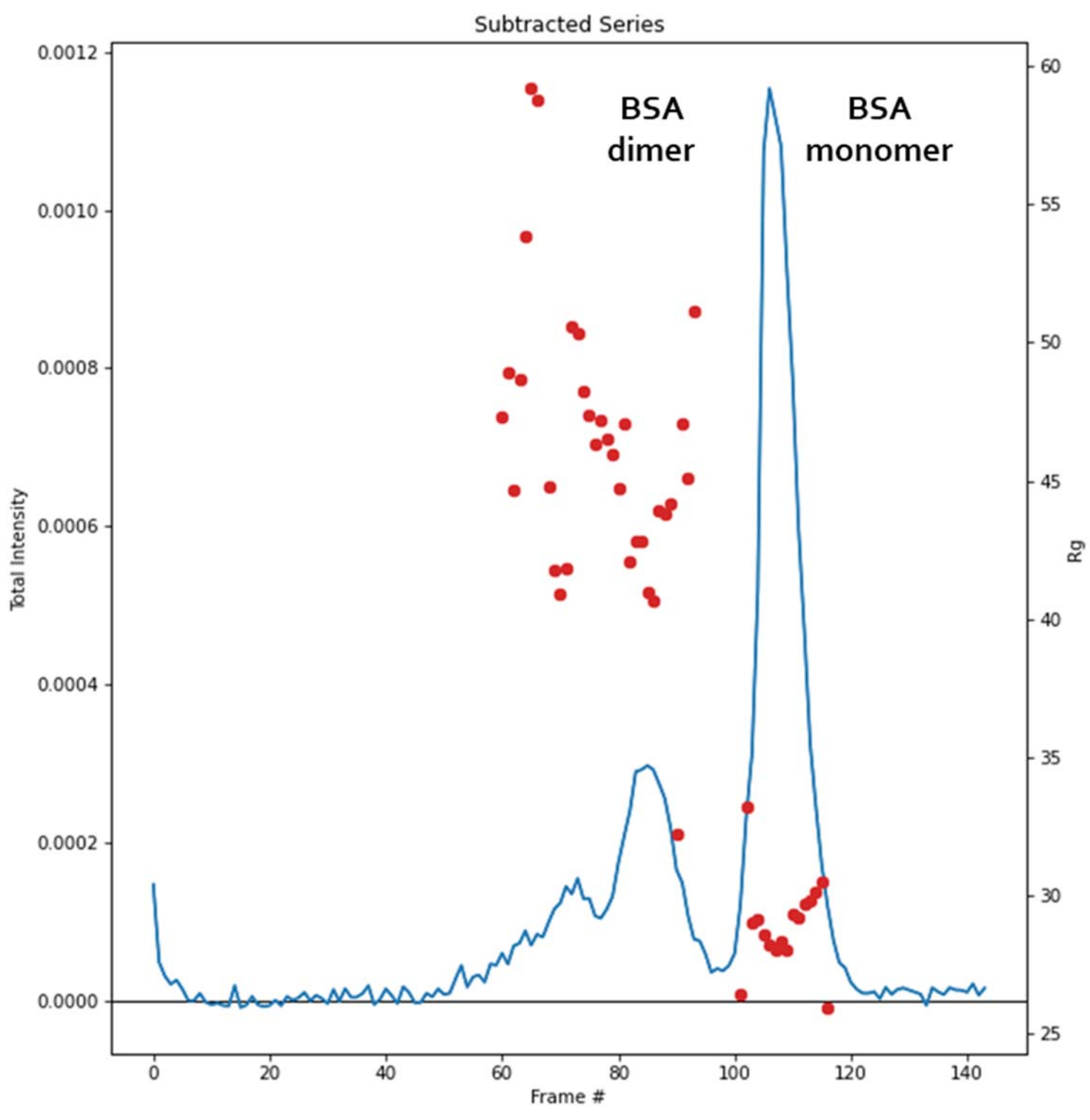

Figure S4. Integrated SAXS signal as a function of frame for the monomer and dimer species of BSA, obtained using Raw (Hopkins et al. 2017)

References

Hopkins, J. B., Gillilan, R. E., & Skou, S. (2017). BioXTAS RAW: improvements to a free open-source program for small-angle X-ray scattering data reduction and analysis. Journal of Applied Crystallography, 50(5), 1545–1553. https://doi.org/10.1107/S1600576717011438
